# The iHealth-T2D study: a cluster randomised trial for the prevention of type 2 diabetes amongst South Asians with central obesity and prediabetes—a statistical analysis plan

**DOI:** 10.1186/s13063-022-06667-1

**Published:** 2022-09-06

**Authors:** Mirthe Muilwijk, Marie Loh, Sara Mahmood, Saranya Palaniswamy, Samreen Siddiqui, Wnurinham Silva, Gary S. Frost, Heather M. Gage, Marjo-Riitta Jarvelin, Ravindra P. Rannan-Eliya, Sajjad Ahmad, Sujeet Jha, Anuradhani Kasturiratne, Prasad Katulanda, Khadija I. Khawaja, Jaspal S. Kooner, Ananda R. Wickremasinghe, Irene G. M. van Valkengoed, John C. Chambers

**Affiliations:** 1 Department of Public Health, Amsterdam UMC, University of Amsterdam, Amsterdam Public Health Research Institute, Meibergdreef 9, Amsterdam, The Netherlands; 2 Amsterdam Public Health, Health Behaviours & Cardiovascular Diseases, Amsterdam Cardiovascular Sciences, Diabetes & Metabolism, Amsterdam, The Netherlands; 3 Lee Kong Chian School of Medicine, Nanyang Technological University, Singapore, 308232 Singapore; 4 Department of Epidemiology and Biostatistics, School of Public Health, Imperial College London, St Mary’s Campus, Norfolk Place, London, W2 1PG UK; 5 Department of Endocrinology & Metabolism, Services Institute of Medical Sciences, Services Institute of Medical Sciences, Services Hospital, Ghaus Ul Azam, Jail Road 54700, Lahore, Pakistan; 6 Center for Life Course Health Research, Faculty of Medicine, University of Oulu, Oulu, Finland; 7 Max Healthcare, Institute of Endocrinology, Diabetes and Metabolism, Max Super Speciality Hospital, 2, Press Enclave Road, Skaet, New Delhi 110017 India; 8 Faculty of Medicine, Imperial College London, Hammersmith Campus, DuCane Road, London, W12 ONN UK; 9 Department of Clinical and Experimental Medicine, Surrey Health Economics Centre, University of Surrey, Leggett Building, Daphne Jackson Road, Guildford, Surrey, GU2 7WG UK; 10 Unit of Primary Care, Oulu University Hospital, University of Oulu, Oulu, Finland; 11 Department of Life Sciences, College of Health and Life Sciences, Brunel University London, Kingston Lane, Uxbridge, UB8 3PH Middlesex UK; 12 Institute for Health Policy, 72 Park Street, Colombo, 00200 Sri Lanka; 13 Punjab Institute of Cardiology, Punjab Institute of Cardiology, Jail Road, Shadman, Lahore, Punjab, Pakistan; 14 Faculty of Medicine, University of Kelaniya, Thalagolla Road, PO Box 06, Ragama, 11010 Sri Lanka; 15 Department of Clinical Medicine, Faculty of Medicine, University of Colombo, 25 Kynsey Rd, Colombo, 00800 Sri Lanka; 16 London Northwest University Healthcare NHS Trust, Uxbridge Road, Southall, UB1 3HW Middlesex UK; 17 National Heart and Lung Institute, Imperial College London, Hammersmith Campus, DuCane Road, London, W12 ONN UK

**Keywords:** Type 2 diabetes, South Asian, Lifestyle intervention

## Abstract

**Background:**

South Asians are at high risk of type 2 diabetes (T2D). Lifestyle modification is effective at preventing T2D amongst South Asians, but the approaches to screening and intervention are limited by high costs, poor scalability and thus low impact on T2D burden. An intensive family-based lifestyle modification programme for the prevention of T2D was developed. The aim of the iHealth-T2D trial is to compare the effectiveness of this programme with usual care.

**Methods:**

The iHealth-T2D trial is designed as a cluster randomised controlled trial (RCT) conducted at 120 sites across India, Pakistan, Sri Lanka and the UK. A total of 3682 South Asian men and women with age between 40 and 70 years without T2D but at elevated risk for T2D [defined by central obesity (waist circumference ≥ 95 cm in Sri Lanka or ≥ 100 cm in India, Pakistan and the UK) and/or prediabetes (HbA1c ≥ 6.0%)] were included in the trial. Here, we describe in detail the statistical analysis plan (SAP), which was finalised before outcomes were available to the investigators. The primary outcome will be evaluated after 3 years of follow-up after enrolment to the study and is defined as T2D incidence in the intervention arm compared to usual care. Secondary outcomes are evaluated both after 1 and 3 years of follow-up and include biochemical measurements, anthropometric measurements, behavioural components and treatment compliance.

**Discussion:**

The iHealth-T2D trial will provide evidence of whether an intensive family-based lifestyle modification programme for South Asians who are at high risk for T2D is effective in the prevention of T2D. The data from the trial will be analysed according to this pre-specified SAP.

**Ethics and dissemination:**

The trial was approved by the international review board of each participating study site. Study findings will be disseminated through peer-reviewed publications and in conference presentations.

**Trial registration:**

EudraCT 2016–001,350-18. Registered on 14 April 2016. ClinicalTrials.gov NCT02949739. Registered on 31 October 2016.

**Supplementary Information:**

The online version contains supplementary material available at 10.1186/s13063-022-06667-1.

## Introduction

Type 2 diabetes (T2D) is the fifth leading cause of death worldwide [1] and a major contributor to the development of various comorbidities including coronary heart disease, stroke, peripheral vascular disease and end-stage renal failure [2]. South Asians, who represent one-quarter of the world’s population, are at high risk of T2D and its complications, both in the country of origin and after migration [3, 4]. Key modifiable risk factors that could be targeted to delay or prevent the onset of T2D include behavioural factors such as diet and physical activity [5]. In the past decades, evidence from the “Finnish Diabetes Prevention Study” and the “Diabetes Prevention Program” showed that targeting these behavioural factors may be effective to delay or prevent the onset of T2D [6, 7]. A recent meta-analysis on 1816 participants from six randomised controlled trials (RCTs) (four from Europe and two from India) has reported lifestyle modifications may also be effective amongst South Asian populations [8].

Although the studies conducted to date provide some support for the utility of lifestyle interventions for the prevention of T2D amongst South Asians, there are significant limitations. First, completed studies conducted in India and Sri Lanka are limited to local settings and small sample sizes, and there are no studies reported from Pakistan. Second, the evidence-based approach established by studies to prevent T2D amongst South Asians lacks scalability and sustainability for T2D prevention, especially in low-middle income settings, since previous lifestyle interventions to prevent T2D were designed in a way that makes them labour-intensive and costly. To address these important limitations, we designed the iHealth-T2D trial in a way that makes the intervention scalable and sustainable in both low-middle income and high-income settings. The iHealth-T2D intervention aims to identify participants as being at risk for T2D based on parameters which include low-resource strategies such as waist circumference and to improve cost-effectiveness and scalability of lifestyle modification through the use of community health workers and a family-based lifestyle modification. Furthermore, we aimed to evaluate the effectiveness of the intervention in different cultural groups living in India, Pakistan, Sri Lanka and the UK, to improve generalisability. The objective of the iHealth-T2D trial is to investigate whether our family-based lifestyle modification delivered by community health workers is effective to prevent T2D amongst South Asians at high risk for T2D (based on central obesity or prediabetes), compared to usual care.

Here, we report the details of the statistical analysis plan (SAP), prepared according to the published guidelines on the content of SAPs [9]. This SAP includes details on the analyses of the primary objective but does not include details on secondary questions nor the evaluation of cost-effectiveness. The cluster RCT is registered with EudraCT 2016–001,350-18 and ClinicalTrials.gov NCT02949739. This SAP should be read in conjunction with the study protocol, which contains more details on the study rationale and design. The study protocol will be published and is until then available upon reasonable request.

## Summary study design

**Fig. 1:**
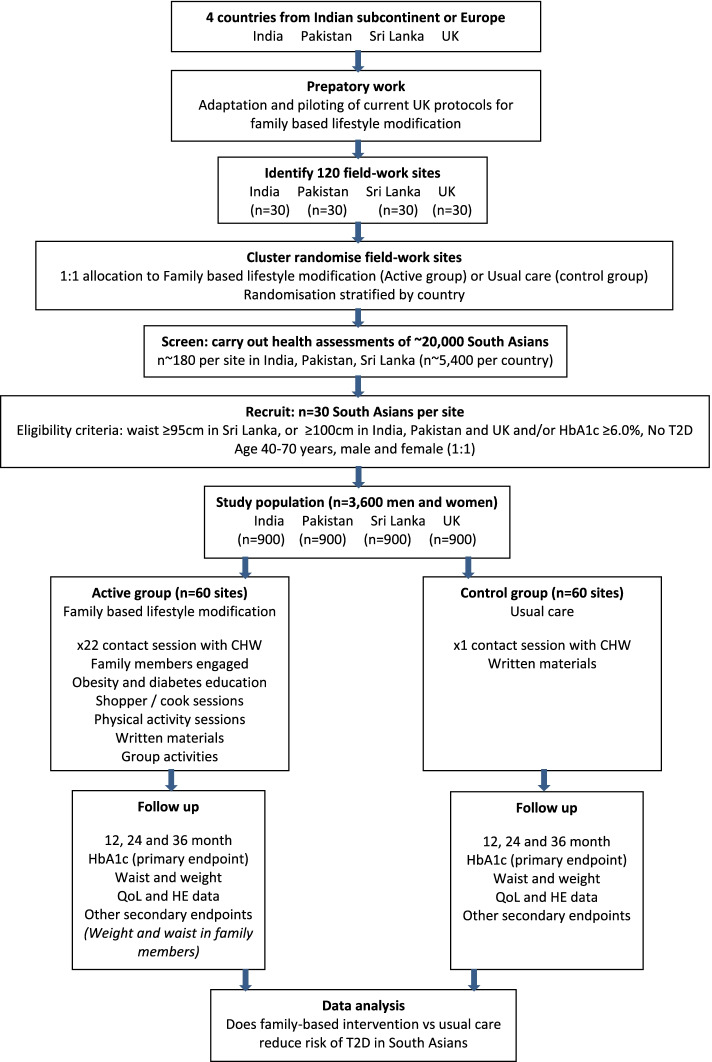
Study flow

The iHealth-T2D trial is designed as a cluster RCT amongst 3682 South Asians at high risk for T2D at 120 fieldwork sites across India, Pakistan, Sri Lanka and the UK. The study design is summarised in Fig. 1; in brief, a total of 120 sites from a range of socio-economic settings were identified, comprising 30 sites in each of the four participating countries (India, Pakistan, Sri Lanka and the UK). Randomisation was conducted by the Imperial College London programme coordinator, pre-recruitment of participants and with no knowledge of any on-the-ground conditions. The fieldwork sites were cluster randomised by computer-generated random numbers stratified by country, to either family-based lifestyle modification or usual care (1:1 allocation). Cluster randomisation was used as it reduces the risk of resentful demoralisation (contamination) during an unblinded intervention [10]. At each fieldwork location, we aimed to recruit 15 male and 15 female South Asians between the ages of 40 and 70 years old, at high risk, but free from T2D. High risk for T2D was defined by central obesity (waist circumference ≥ 95 cm in Sri Lanka or ≥ 100 cm in India, Pakistan and the UK) and/or prediabetes (HbA1c ≥ 6.0%). The exclusion criteria included participants with known type 1 or type 2 diabetes, fasting glucose levels ≥ 7.0 mmol/L, HbA1c levels ≥ 6.5%, BMI < 22 kg/m^2^, pregnant or planning pregnancy, unstable residence or planning to relocate and serious illness.

The primary aim of the lifestyle modification sessions was the prevention of T2D, and details on the various sessions can be found in the iHealth-T2D study protocol. In brief, participants in the lifestyle modification arm received family-based lifestyle modification delivered by a community health worker, consisting of 22 contact sessions over a period of 12 months. Participants in the usual care arm received a single diabetes prevention education session lasting 30–60 min delivered by a community health worker. Written material was distributed additionally. The usual care group received no further treatment from the research team and received their care as they would usually have received it. Both participants and community health workers could not be blinded to the treatment arm as the type of care given is clearly visible. They were, however, kept masked to the outcome measurements and trial results. Participants were followed up annually, during a period of 3 years. Data were obtained by research nurses who were blinded to the trial arms to reduce the risk of assessment bias.

Ethical approval was obtained from the Institutional Review Board in each participating country and at each research location before the start of the study. Information sheets and consent forms were made available in the major South Asian languages. Multilingual translators were available as required. Each participant provided informed consent. People unwilling or unable to provide consent were excluded from the study. The research complied with relevant national and international regulations and was carried out in line with the Declaration of Helsinki and the International Conference on Harmonization Guidelines.

## Study outcomes

The effectiveness of the iHealth-T2D intervention will be evaluated at two time points, both after 1 and after 3 years of follow-up. After 1 year of follow-up, secondary outcomes are evaluated, while after 3 years of follow-up, both the primary outcome and secondary outcomes will be evaluated.

### Primary outcome

The primary outcome is defined as follows: T2D incidence in the intervention arm compared to the usual care arm after 3 years of follow-up in both groups of South Asians with central obesity and prediabetes. T2D incidence is defined as a physician diagnosis and being on treatment for T2D or HbA1c levels ≥ 6.5% [11]. The primary outcome will not be evaluated after 1 year of follow-up as power calculations were based on a 3-year follow-up duration, and the power for this dichotomous outcome variable will be too low after 1 year of follow-up.

### Secondary outcomes

Secondary outcomes include glucose, insulin, homeostatic model assessment (HOMA), total cholesterol, HDL cholesterol, triglycerides, blood pressure, waist circumference, weight, smoking status, alcohol use, physical activity, dietary intake, treatment compliance and dose delivered/received and are, therefore, evaluated both after 1 and 3 years of follow-up. The evaluation of the secondary outcomes after 1 as well as 3 years of follow-up will allow for the comparisons of both short- and long-term effects of the lifestyle intervention. In addition, subgroup analyses amongst participants included in the study based on HbA1c and/or waist circumference will be performed.

### Secondary questions

Study outcomes that will be reported but are not included in the current SAP are secondary questions which include changes in adiposity and glucose homeostasis amongst the extended family, psychosocial measures, cost-effectiveness and implications of scaling up locally and nationally.

### Power calculations

R version 3.6.3. (R project for Statistical Computing) was used for power calculations, with the clusterPower package. The total number of included clusters per study arm in the study is 60, with 30 participants in each cluster. An intraclass correlation coefficient (ICC) of 0.01 was assumed. The findings in previous work report that median ICCs in primary care lay around 0.01 [12, 13], but published trials did not report appropriate ICCs; therefore, we performed power calculations under alternate assumptions of the ICC. Power calculations are based on the overall effects in the total group, with the analyses by subgroup reported as supplementary information. Therefore, the main analyses will not be adjusted for multiple testing, which is in line with the current recommendations [14]. A meta-analysis showed that lifestyle modifications may lead to a T2D risk reduction of 35% [8]. Assuming that event rates for T2D for usual care are 6.8% per year [15], a two-sided significance level of 5% and an ICC of 0.05, the study has 90% power to identify a reduction in T2D incidence of 35% in the intervention compared to the usual care group [8], after a follow-up period of 3 years with an estimated drop-out rate of 10% of the clusters and 10% of the participants in the clusters (Table 1). The study was, thus, sufficiently powered for the primary outcome. Since secondary outcomes are of continuous nature, it is expected that a statistically significant change can already be detected after 1 year of follow-up (Additional file 1).

**Table 1:** The detectable risk reduction for T2D in the intervention compared to the control group. Power was set to 80% and *p* < 0.05. There were a total of 60 clusters with 30 participants in each cluster. Alternate assumptions were compared, e.g. 10% drop-out in clusters and 10 and 20% drop-outs rates in participants and with 20% and 40% lower T2D event rates than estimated. The baseline predicted event rate for the control group was 6.8% per year or 19.0% in 3 years

Assumptions				Detectable risk reduction
**Drop-out rate clusters**	**Event rate**	**Drop-out rate participants**	**ICC**	
0% (*n* = 60)	Baseline (19.0% in 3 years)	0% (*n* = 30/cluster)	0.01	21%
0% (*n* = 60)	Baseline (19.0% in 3 years)	0% (*n* = 30/cluster)	0.05	28%
0% (*n* = 60)	Baseline (19.0% in 3 years)	0% (*n* = 30/cluster)	0.1	35%
0% (*n* = 60)	Baseline (19.0% in 3 years)	10% (*n* = 27/cluster)	0.01	22%
0% (*n* = 60)	Baseline (19.0% in 3 years)	10% (*n* = 27/cluster)	0.05	29%
0% (*n* = 60)	Baseline (19.0% in 3 years)	10% (*n* = 27/cluster)	0.1	36%
0% (*n* = 60)	Baseline (19.0% in 3 years)	20% (*n* = 24/cluster)	0.01	23%
0% (*n* = 60)	Baseline (19.0% in 3 years)	20% (*n* = 24/cluster)	0.05	30%
0% (*n* = 60)	Baseline (19.0% in 3 years)	20% (*n* = 24/cluster)	0.1	36%
10% (*n* = 54)	Baseline (19.0% in 3 years)	10% (*n* = 27/cluster)	0.01	22%
10% (*n* = 54)	Baseline (19.0% in 3 years)	10% (*n* = 27/cluster)	0.05	30%
10% (*n* = 54)	Baseline (19.0% in 3 years)	10% (*n* = 27/cluster)	0.1	37%
0% (*n* = 60)	20% lower (15.2% in 3 years)	10% (*n* = 27/cluster)	0.01	24%
0% (*n* = 60)	20% lower (15.2% in 3 years)	10% (*n* = 27/cluster)	0.05	32%
0% (*n* = 60)	20% lower (15.2% in 3 years)	10% (*n* = 27/cluster)	0.1	40%
0% (*n* = 60)	40% lower (11.4% in 3 years)	10% (*n* = 27/cluster)	0.01	28%
0% (*n* = 60)	40% lower (11.4% in 3 years)	10% (*n* = 27/cluster)	0.05	37%
0% (*n* = 60)	40% lower (11.4% in 3 years)	10% (*n* = 27/cluster)	0.1	46%

## Statistical analysis plan

### General principles

The analysis of the primary outcome will be performed after 3 years of follow-up. The secondary outcomes will be evaluated both after 1 and 3 years of follow-up. Analyses will be performed by the investigators of the iHealth-T2D study group (MM for the 1-year analyses), who were blinded for the intervention, on a clean anonymised data set (JCC and AK). The latest version of the R statistical software package will be used. Tests will be two-sided, and *p*-values < 0.05 will be considered statistically significant. All statistical analyses will be adjusted for confounders registered at baseline, namely age and sex. We will not adjust for multiple testing as we pre-defined the primary and secondary outcomes [14]. Data will be reported in line with the Consolidated Standards of Reporting Trials (CONSORT) 2010 statement: extension to cluster randomised trials [16].

The analyses will be performed according to the intention-to-treat (ITT) principle with data from all clusters and participants enrolled in the study [17, 18]. Data of participants who attended at least one post-baseline assessment will be analysed according to their initially assigned study arm, regardless of their adherence. Participants who withdrew their consent will be excluded from the ITT analyses, and the number of participants who withdrew their consent and reasons for withdrawing consent will be reported. The patterns of missing data for the primary and secondary outcomes and, if known, reasons for missingness will be summarised for both treatment arms [19]. The nature and pattern of missing data will be explored. If data missing at random is assumed [20], data will be imputed by multiple imputation methods and primary analysis will be performed with the imputed data. If data is not missing at random, a “best case/worst case” sensitivity analysis will be used [21]. In case multiple imputation is used for one or more outcomes, we will use the jomo wrapper in the mice package in the R software for statistical analyses; variables selected as predictors for imputation contain known predictors of T2D. These include covariates included in the main analysis model (sex, age) and the auxiliary variables country, setting, socio-economic status, pack-years of smoking, alcohol consumption, metabolic equivalents (METs) of physical activity, waist circumference and HbA1c. Missing values will be imputed separately by the allocated randomisation group [22] and will comply with the multi-level character of the data.

### Baseline characteristics

Baseline characteristics of both study arms will be presented by sex and country and presented in a table. The baseline characteristics will not be tested for statistical differences between study arms [23]. The baseline characteristics will be reported by arithmetic means and standard deviation (normally distributed numerical data), medians and interquartile ranges (non-normally distributed numerical data) or percentages and numbers (categorical data). Normality of data distributions will be inspected visually by plotting histograms, and we will assess the deviation from normality by the Shapiro–Wilk test. In case of a *p*-value > 0.05, the data will be transformed for normality, before any statistical analyses.

Descriptive characteristics to report at baseline include age (years), setting (%), socio-economic status (%), smoking (pack-years), alcohol consumption (units/week), physical activity (MET/week), BMI (kg/m^2^), waist circumference (cm), HbA1c (%) and glucose (mmol/L). The population size and number of missing observations will also be reported.

### Analyses of the primary outcome

Cumulative incidence of T2D will be summarised and compared between the treatment arms using random effects logistic regression to estimate the odds ratios (OR) and 95% CI. The R package lme4 for generalised linear mixed models will be used, which includes the frequentist method to estimate the fixed and random effects; the default correlation structure is unstructured [24]. In addition, we will evaluate the intraclass correlation coefficients to assess the cluster variance; the R package sj stats, version 0.17.5, will be used. The model will include the randomisation stratum site as a random effect and treatment and country as a fixed effect. The effectiveness of the lifestyle intervention will be reported by the risk difference and the screening numbers needed to identify one case of “high risk” for developing diabetes and the number needed to treat or delay one case of T2D. The risk difference will be derived with the modified log-Poisson approach [25]. The number needed to treat will be derived by the R package nnt, which is based on the restricted mean survival time in the control group divided by the difference in restricted mean survival time between the treatment and control groups up to 3 years of follow-up. The Wilson score method will be used to calculate CIs [26]. All analyses will be adjusted for the confounders age and sex.

### Analyses of the secondary outcomes

The secondary outcomes are of continuous nature and will be reported as mean and SD in each of the two treatment groups. The differences between the two treatment arms will be estimated with a multilevel linear mixed-effects regression model. The models will include the stratification variable country as a fixed effect and a random effect for clusters. The estimates will be presented with their associated 95% confidence intervals (CIs) and *p*-values for comparison between the treatment groups. In addition, adjustments for age and sex will be performed, and the baseline values will be reported.

### Additional analyses

Treatment compliance will be reported for the intervention arm as an explanatory variable. It will be reported according to the number of times a participant turned up for the lifestyle modification (LSM) sessions. In addition, changes in dietary intake will be reported. Twenty-four-hour dietary recalls and food frequency questionnaires were performed in the treatment arm only. Dietary variables will, therefore, be reported as a change from baseline in the treatment arm.

Both absolute and relative risk reduction will be compared for subgroups of participants included in the study based upon a high risk for T2D according to waist circumference measurements (waist circumference ≥ 100 cm in India and Pakistan; ≥ 90 cm in Sri Lanka) and those included based upon HbA1c levels (6.0–6.4% inclusive). The interaction of the treatment arm with sex, setting, socio-economic status, baseline waist circumference and HbA1c levels will be assessed. If there is an interaction, effect estimates and *p*-values will be presented by subgroups.

Sensitivity analyses will be performed to identify potentially extreme sites, because the extreme deviation of one site from other sites may have a large impact on the overall results. This is done by leaving one site out at a time; a centre is considered extreme as the estimate changes by > 10%. In addition, complete case analyses will be conducted to assess the robustness of the results.

Since lifestyle interventions are generally considered to be safe, no (serious) adverse events are to be expected. In case of any adverse events, these will be reported per incident with the number per group and a description of the event.

## Discussion

The iHealth-T2D cluster RCT will provide evidence whether an intensive family-based lifestyle modification programme delivered by community health workers compared to usual care is effective to prevent T2D amongst South Asians at high risk for T2D based on central obesity or prediabetes and living in India, Pakistan, Sri Lanka and the UK. Here, we have provided details of the planned statistical analyses of the iHealth-T2D cluster RCT and pre-specified primary and secondary outcomes, both after 1 and 3 years of follow-up, together with planned analyses. Statistical decisions may influence the final conclusions of the intervention’s effectiveness. Reporting the SAP before commence of analyses increases transparency [9] and reduces the risk of bias by outcome reporting and data-driven analyses [27]. This SAP contains details on all elements of the statistical analyses limiting the risk of bias, e.g. by reporting only the outcomes for which a statically significant effect was identified [27].

Although statistical methods are chosen as objectively as possible, there is always unavoidable subjectivity involved. In addition, multiple perspectives may be relevant to each statistical discussion which may lead to different choices. In addition, chosen thresholds may be somewhat arbitrary. An example is the common use of *p*-values which is currently under debate [28]. We will report *p*-values and label < 0.05 as statistically significant but will also consider the effect sizes to help identify the effectiveness of the trial. Reporting a statistical analysis plan will help to counteract selection bias considering not reporting the study results above the *p*-value threshold, since it promotes the publication of non-significant findings [29]. This will make the cumulative evidence of multiple individual studies more reliable.

The analyses will be based on ITT, which is currently considered as the gold standard for RCTs [17]. In ITT-based analyses, subjects that did not comply with the assigned treatment of the study arm or dropped out of the study are still included in the analyses according to their assigned study arm. A drawback of this approach is that the effect size of the treatment will thus be underestimated, and results may be more susceptible to type II errors. In addition, interpretability might become difficult since dose–response is unclear. An advantage of the ITT is that participants that are less likely to comply with the intervention and are thus more likely to drop out are still included in the study results. The ITT approach will thus give study results that take the likeliness of adopting the lifestyle intervention in the general population into account. Altogether, the analyses based on ITT will be conservative, but mostly unbiased since the balance in participants generated by the random treatment allocation is maintained.

Multiple imputation of data, including outcome data, is recommended in case data is missing at random, when over 5% and less than 40% of the data is missing, and if auxiliary variables are identified [30]. However, none of the statistical techniques currently available can completely compensate for the lack of true data. In addition, the estimates of treatment effect only remain unbiased in case the analysis model is correctly specified [22]. Bias is minimised if imputation is carried out separately by the randomisation group. This approach may, however, be less conservative. Participants with missing data are more likely to be non-compliant with the lifestyle intervention, while imputed data may reflect those without missing data in the treatment arm. Other approaches to deal with missing data include the last observation carried forward and complete-case analysis, but both are sensitive to generating biassed estimates, and we therefore did not consider these approaches [31].

In conclusion, providing the details of the SAP for the iHealth-T2D trial will help to minimise bias in the publication of our study outcomes. The selected statistical methods were based on the current consensus on the most appropriate methods according to scientific literature but are always under debate. Sensitivity analyses will, therefore, include conservative estimates of the effect of the iHealth-T2D trial. If the iHealth-T2D intervention is proven effective, this family-based lifestyle modification is designed in a way that it may be used in a wide range of settings, including those with a low availability of resources, to prevent T2D amongst South Asians.

## Trial status

Version: 1.0, date: February 7, 2020.

The first participant was enrolled on 15/06/2016 and the last participant on 05/03/2019. The last scheduled follow-up date is 05/03/2022. The SAP has been submitted after the end of recruitment as it is submitted in conjunction with the study protocol, which was not ready in time. The study protocol is in line with the funding application. This document has been written based on the information contained in the Clinical Study Protocol version 2, dated 01/11/2016.

SAP revision history:

**Table:** 

Protocol version	Updated SAP version no	Section number changed	Description of and reason for change	Date changed

## Supplementary Information

**Additional file 1.** Detectable difference in secondary study outcomes after one year of follow-up.

## Data Availability

Data will be available to others on completion of the research, by application to the Steering Committee.

## Abbreviations

CI: Confidence interval
CONSORT: Consolidated Standards of Reporting Trials
CRT: Controlled randomised trial
ICC: Intraclass correlation coefficient
ITT: Intention-to-treat
LSM: Lifestyle modification
MET: Metabolic equivalents
OR: Odds ratio
SAP: Statistical analysis plan
SD: Standard deviation
T2D: Type 2 diabetes
