# Supplementary material for: The iHealth-T2D study: a cluster randomised trial for the prevention of type 2 diabetes amongst South Asians with central obesity and prediabetes—a statistical analysis plan
Source: Trials. 2022 Sep 6;23:755. doi: 10.1186/s13063-022-06667-1 (PMC9450360; doi:10.1186/s13063-022-06667-1)
Supplement: Supplementary file 1 — Additional file 1. Detectable difference in secondary study outcomes after one year of follow-up. [file 13063_2022_6667_MOESM1_ESM.pdf]

Supplementary information

Additional file 1: Detectable difference in secondary study outcomes after one year of follow-up.

		**Detectable difference**		
**Measure**	**Expected SD**	**0% Dropout**	**10% Dropout**	**20% Dropout**
Waist (cm)	11.5	1.23	1.28	1.34
Weight (kg)	7.5	0.80	0.84	0.88
Glucose (mmol/L)	0.6	0.06	0.07	0.07
HbA1c (%)	0.5	0.05	0.06	0.06
HOMA	3.0	0.32	0.33	0.35
Total cholesterol (mmol/L)	0.9	0.10	0.10	0.11
Triglycerides (mg/dL)	1.9	0.20	0.21	0.22
Blood pressure systolic (mmHg)	16	1.71	1.78	1.87
Blood pressure diastolic (mmHg)	8.6	0.92	0.96	1.00
Dietary intake (Kcal)	390	41.7	43.4	45.5

Expected detectable difference for a range of secondary study outcomes after 1 year of follow-up, with a total of 60 clusters per study arm and 30 participants in each cluster, assuming i) standard deviation (SD) of 10 cm with a two-sided 5% significance level and ii) ICC of 0.01. iii) a two sided p-value of 0.05 iv) a power of 0.80.
